# A Systematic Review of Ongoing Registered Research Studies on Post-Operative Atrial Fibrillation after Cardiac Surgery

**DOI:** 10.3390/jcm13164948

**Published:** 2024-08-22

**Authors:** Ivy Quan, Emilie P. Belley-Côté, Jessica Spence, Austine Wang, Karen Sidhom, Michael Ke Wang, David Conen, Bryan Sun, Aadithya Udaya Shankar, Richard P. Whitlock, P. J. Devereaux, Jeff S. Healey, William F. McIntyre

**Affiliations:** 1 Faculty of Health Sciences, McMaster University, Hamilton, ON L8L 2X2, Canada; ivy.quan@mail.utoronto.ca (I.Q.); austine.wang@medportal.ca (A.W.); ksidhom2@uwo.ca (K.S.); bryan.sun@medportal.ca (B.S.); aadithya.udayashankar@medportal.ca (A.U.S.); 2 Population Health Research Institute, Hamilton, ON L8L 2X2, Canada; emilie.belley-cote@phri.ca (E.P.B.-C.); jessica.spence@phri.ca (J.S.); david.conen@phri.ca (D.C.); richard.whitlock@phri.ca (R.P.W.); pj.devereaux@phri.ca (P.J.D.); jeff.healey@phri.ca (J.S.H.)

**Keywords:** post-operative atrial fibrillation, cardiac surgery, perioperative medicine, stroke, rate control, rhythm control, oral anticoagulation

## Abstract

**Background/Objectives:** New-onset atrial fibrillation (AF) after cardiac surgery is associated with patient-important outcomes. Uncertainty persists regarding its prevention, detection, and management. This review seeks to identify, compile, and describe ongoing registered research studies involving patients with or at risk for post-operative AF (POAF) after cardiac surgery. **Methods:** We searched clinical trial registries in January 2023 for studies focusing on POAF prediction, prevention, detection, or management. We extracted data from each record and performed descriptive analyses. **Results:** In total, 121 studies met the eligibility criteria, including 82 randomized trials. Prevention studies are the most common (*n* = 77, 63.6%), followed by prediction (*n* = 21, 17.4%), management (*n* = 16, 13.2%), and detection studies (*n* = 7, 5.8%). POAF after cardiac surgery is an area of active research. **Conclusions:** There are many ongoing randomized prevention studies. However, two major clinical gaps persist; future randomized trials should compare rate and rhythm control in patients who develop POAF, and long-term follow-up studies should investigate strategies to monitor for AF recurrence in patients with POAF.

## 1. Introduction

Atrial fibrillation (AF) is the most common clinical arrhythmia, and a frequent complication of cardiac surgery. Post-operative AF (POAF) carries important health and economic burdens. Its incidence depends on the type of cardiac surgery and method of detection, with the highest rates (50–62%) reported in patients undergoing combined valve surgery and coronary artery bypass grafting (CABG) [1,2]. POAF is associated with a two- to four-fold increased risk of stroke in the 30 days after surgery [1,3]. Patients with POAF spend 4–5 days more in hospital at a cost of more than $10,000 USD per patient [1,4,5]. POAF is associated with higher in-hospital mortality (odds ratio [OR]: 1.7, 95% confidence interval (CI): 1.25–2.25) and long-term mortality (OR: 3.4, 95% CI: 1.58–7.45) compared to patients without POAF [5]. Clinicians are interested in identifying the highest-risk patients and preventing POAF in as many patients as possible.

One key unresolved issue in the treatment of patients who develop POAF is the choice between rate and rhythm control. The 2020 European Society of Cardiology (ESC) guidelines report that the decision between rate and rhythm control should be based on patient symptoms [6]. The 2020 Canadian Cardiovascular Society (CCS) guidelines recommend either a rate or a rhythm control strategy (strong recommendation, moderate-quality evidence) [7]. The 2023 American Heart Association (AHA) guidelines corroborate this, and recommend either rate or rhythm control for hemodynamically stable patients (Class I, Level B—with RCT data). For hemodynamically unstable patients, they recommend direct current cardioversion alongside rhythm control [8]. The 2017 European Association for Cardiothoracic Surgery recommends rhythm control for asymptomatic, hemodynamically stable patients (Class I, Level B), and the consideration of rate control (Class IIa, Level B) [9]. The 2020 ESC, 2020 CCS, and 2023 AHA guidelines all refer to the landmark 2016 RCT by Gillinov et al., which randomized 523 patients with POAF to rate or rhythm control, and found no clinical advantage associated with either method [6,7,8,10]. Based on this study, all three organizations leave the choice between rate and rhythm control up to physician’s judgment.

The use of both short-term and long-term prophylaxis of stroke with oral anticoagulants is another key issue. The 2020 ESC guidelines state that long-term oral anticoagulation may be considered in patients with POAF after cardiac surgery considering the anticipated net clinical benefit and informed patient preferences; however, this is only a Class IIb recommendation with Level B evidence [6]. The 2023 AHA guidelines state that administering anticoagulation is reasonable in the absence of surgical bleeding for up to 60 post-operative days, and that the need for long-term OAC should be re-evaluated then (Class IIa, Level B—with no RCT data) [8]. These recommendations are based primarily on observational studies, mostly from administrative data [11]. In terms of monitoring, the CCS recommends that patients with transient POAF be followed indefinitely for the emergence of clinical AF, but does not provide guidance on how to monitor these patients [7]. A few small studies have been completed that suggest high rates of AF recurrence with implanted monitors [12,13]. Despite the widespread availability of multiple technologies for rhythm monitoring, there is no consensus approach to differentiating between reversible POAF and recurrent AF indicating persistent or paroxysmal AF.

Given the importance of POAF to patients, clinicians, and healthcare systems, it is an area of ongoing research. Researchers, funding bodies, and professional societies should be aware of ongoing research studies so that they can anticipate which knowledge gaps will persist in the years ahead. In this way, they can be sure the studies that they design, fund, and support will still be needed at the time of their completion.

We undertook a systematic review to describe ongoing registered studies of patients with or at risk for POAF. By comprehensively describing the research landscape, we aimed to identify upcoming potential therapies and identify knowledge gaps that may need to be addressed in future studies.

## 2. Materials and Methods

We pre-registered the study protocol with Open Science Frameworks [14]. We amended the protocol to proceed without risk of bias assessment due to the lack of information for ongoing studies present in clinical trial registries. A second amendment to the protocol is the inclusion of studies captured from 2017 to 19 January 2023 instead of from 2017 to 2022 because we conducted the searches on 19 January 2023.

We searched Clinicaltrials.gov and the World Health Organization’s International Clinical Trials Registry Platform (WHO ICTRP) (which includes ANZCTR, ChiCTR, Clinicialtrials.gov, CTRI, Dutch Trial Registry, EUCTR, IRCT, ISRCTN, and UMIN-CTR) over the last five years (i.e., 1 January 2017 to 19 January 2023) using the search terms cardiac surgery or cardiovascular surgery or valvular surgery or coronary artery bypass graft and atrial fibrillation or atrial flutter. We imported the search results into the Covidence software (Veritas Health Innovation, Melbourne, VIC, Australia). Reviewers assessed the summary of each search record independently and in duplicate, and if either reviewer thought a study could be eligible, it then underwent a full review. In duplicate, we reviewed the full records for items deemed potentially relevant based on their title and abstract summary, and the senior review author resolved disagreements through discussion. For registered studies with a “completed” status or for those that had not been updated in more than one year, the reviewers searched MEDLINE, Embase, Cochrane Central, and the trial registries for an associated publication or available final results.

We included studies that met the following criteria:

1. Participants were undergoing or had undergone cardiac surgery via sternotomy or a minimally invasive surgical approach.
2. Participants were aged 18 or above.
3. POAF was used to define the study population or evaluated as a primary outcome.
4. The study status in the registry record was most recently updated on 1 January 2017, or later.

**Figure 1:**
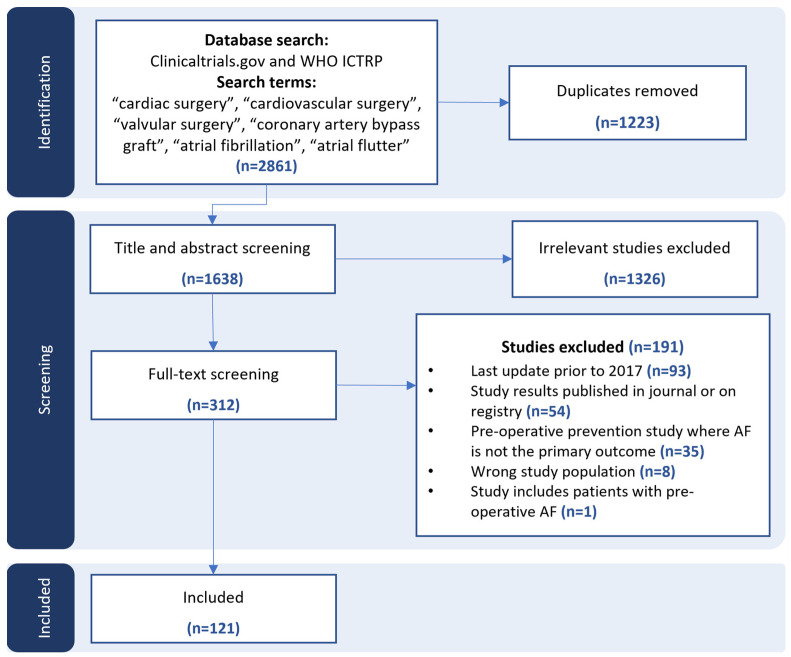
PRISMA flow diagram of search strategy and study selection.

We did not place any limitation on the study design; we included both randomized and nonrandomized studies. We excluded studies with published results and those that included patients with a pre-operative diagnosis of paroxysmal, persistent, or permanent AF (Figure 1).

Independently and in duplicate, the reviewers extracted the registry and registry number, registration year and last update, study status, study theme, study design, planned sample size, geographic location, intervention or comparator, main predictor, primary outcome, and outcome/study timeframe. We recorded the actual sample size for studies that had completed enrolment; otherwise, we recorded the planned sample size.

**Figure 2:**
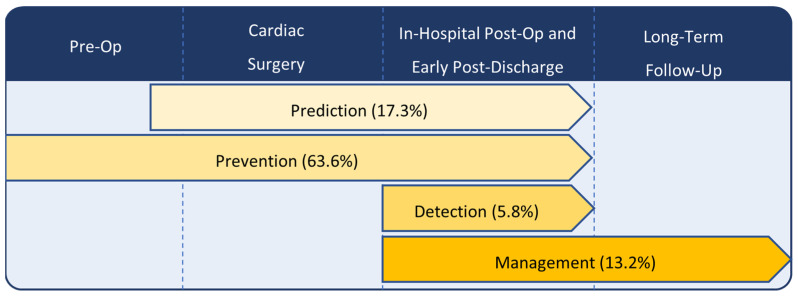
Study themes of ongoing POAF studies throughout the cardiac perioperative journey.

Two reviewers classified the studies into four categories: prediction, prevention, detection, and management. Figure 2 summarizes how these study themes align during the perioperative patient journey. Prediction studies use an observational design, identify at-risk patients before cardiac surgery, and examine the association of one or more predictors with the occurrence of AF. Prevention studies identify or enroll patients before surgery and use an observational or randomized design to test whether an intervention decreases the incidence of AF. Detection studies use an observational or randomized design to test the efficacy of a modality for diagnosing AF in the post-operative period. Management studies use an observational or randomized design to test an intervention to prevent adverse outcomes in patients who develop POAF. We further classified management studies into three themes: rate versus rhythm control, antithrombotic strategies, and monitoring for AF recurrence beyond the perioperative period.

## 3. Results

Figure 1 summarizes the screening and study selection process. After screening 1638 abstracts, we retrieved 312 for full-text review and included 121 studies. Over half (*n* = 68, 56.2%) of the included studies originated from Clinicaltrials.gov, while the other most frequent registries were the Iranian Registry of Clinical Trials (*n* = 17, 14.0%) and the Chinese Clinical Trial Register (*n* = 11, 9.1%) via the WHO ICTRP. See Table A1 in Appendix A for a further study demographic breakdown.

**Figure 3:**
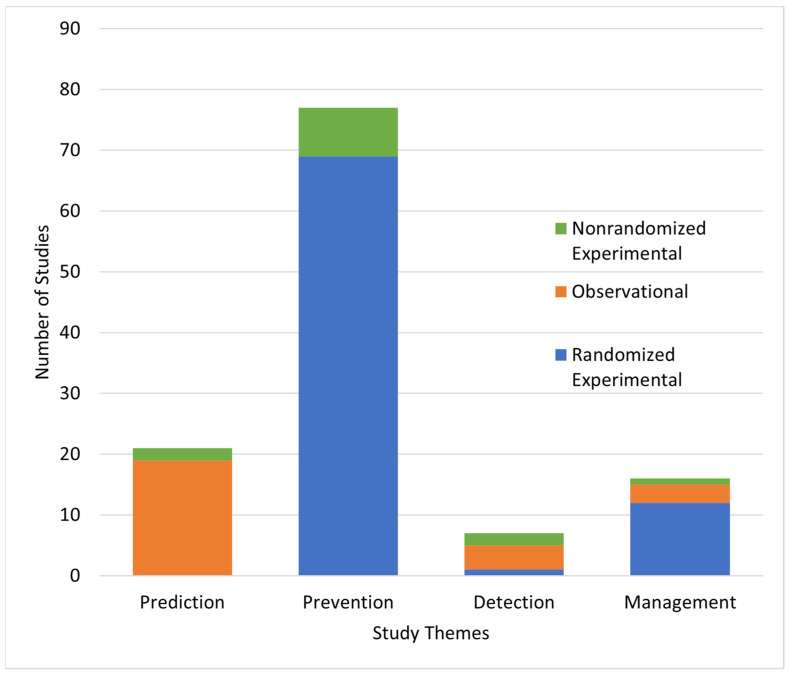
Distribution of study themes and designs in ongoing studies of POAF in cardiac surgery.

Figure 2 describes the temporal association between the four study themes, and Figure 3 describes the distribution of these themes and study designs. Most studies focus on POAF prevention (*n* = 77, 63.6%), while the remaining studies focus on prediction (*n* = 21, 17.4%), management (*n* = 16, 13.2%), and detection (*n* = 7, 5.8%). All studies look at a single study theme except for one, which investigates a combination of prediction and prevention strategies. We categorized this study under prevention in accordance with its main focus (NCT03868150) [15]. Of the 121 included studies, 67.8% (*n* = 82) are randomized trials, 21.5% (*n* = 26) are observational studies, and 10.7% (*n* = 13) are nonrandomized experimental studies.

**Figure 4:**
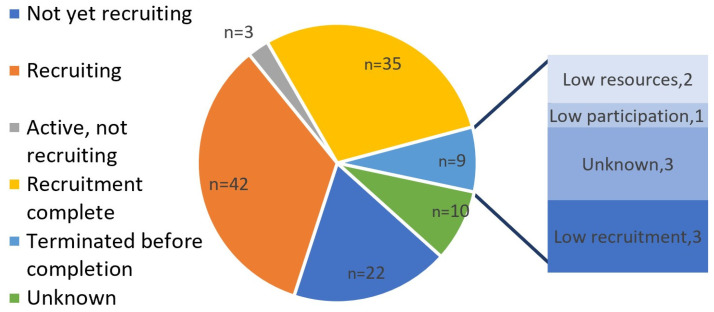
Distribution of study statuses and reasons for termination in ongoing studies of POAF in cardiac surgery.

Figure 4 describes the status of the studies, including the reasons for termination where appropriate. The proportion of studies that are ongoing (Not yet recruiting; Recruiting; Active, not yet recruiting; or Recruitment Complete) is 84.3% (*n* = 102), while 7.4% (*n* = 9) are terminated and 8.3% (*n* = 10) have a an “unknown” status in their registry. Two thirds of terminated or withdrawn studies cited low recruitment or lack of resources as the reason for termination (*n* = 6/9, 66.7%). Among the studies with a current official status of “unknown”, all had a last known official status of “recruiting”.

**Figure 5:**
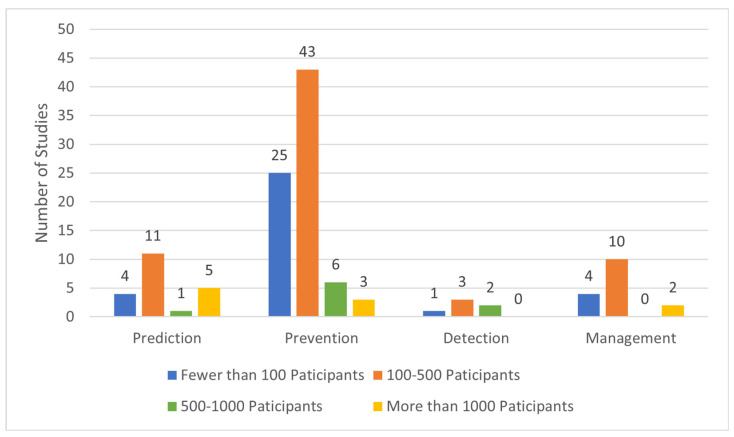
Distribution of sample sizes across themes in ongoing studies of POAF in cardiac surgery.

Figure 5 provides an overview of the relationship between study theme and sample sizes. In prediction studies, the study size ranges from 50 to 25,000 participants, with a median of 170 participants. Prevention studies have a median sample size of 160 participants, ranging from 16 to 1684 participants. The three included prevention studies with more than 1000 participants are randomized trials. The sample size of detection studies ranges from 52 to 730 participants, with a mean of 350 participants. Management studies have sample sizes ranging from 40 to 3500 patients, with a mean of 450. The two included management studies with sample sizes over 1000 are randomized trials.

### 3.1. Prediction

Most prediction studies (*n* = 19/21, 90.5%) are observational, with the remaining 9.5% using nonrandomized experimental designs (Figure 3). Table 1 describes the primary variables in prediction studies, most of which (*n* = 17/21, 81.0%) investigate the association or predictive value of individual predictive variables rather than attempting to create or validate predictive tools.

Slightly less than half (*n* = 9, 42.9%) of the predictive studies evaluate atrial imaging, while about a third (*n* = 6, 28.6%) evaluate biomarkers. Four studies attempt to directly create predictive models, which incorporate combinations of the factors shown in Table 1 [16,17,18,19]. Most of the models in ongoing research focus on patient characteristics (using demographic data, medical history, atrial imaging, etc.), while intra-operative events are less studied. The PARADISE study (NCT05255224) is a large, ongoing retrospective cohort study that aims to develop and validate predictive models using both patient characteristics and intra-operative events [19]. This study uses the United Kingdom General Practice Database (CALIBER) and the American Partners Research Database to investigate new risk factors, develop two multi-modality prediction tools, and test the reliability of their tools on patients in the United Kingdom [19].

### 3.2. Prevention

Most prevention studies (*n* = 69/77, 89.6%) are randomized, while the remaining studies (*n* = 8/77, 10.4%) use nonrandomized experimental designs (Figure 3). Table 2 illustrates the interventions and comparators in prevention studies.

Prophylactic studies most commonly evaluate beta-blockade medications, antiarrhythmic medications, vitamins and electrolyte supplements, and autonomic nervous system suppression. Amiodarone (*n* = 10) and botulinum toxin injections into the epicardial fat pads (*n* = 6) are the most frequently studied individual interventions. Several trials study surgical interventions, injections, or other approaches to autonomic nervous system modulation during surgery. All prevention studies use POAF as a primary outcome, and out of those that recorded outcome timeframes, most evaluate for POAF 1–7 days post-operatively (*n* = 37, 66.1%), followed by 8–30 days post-operatively (*n* = 13, 23.2%), and from 30 days to 1 year post-operatively (*n* = 6, 10.7%).

One study (NCT03868150) combines prediction and prevention strategies. The authors are investigating whether the inducibility of AF predicts POAF and if preventative amiodarone injections in patients with inducible AF decrease POAF [15].

### 3.3. Detection

Table 3 describes the modalities used in detection studies.

Four of the seven studies are observational, two are nonrandomized experimental, and one is randomized (Figure 3). Six of the seven studies in this category, three of which involve continuous monitoring devices, explore continuous and intermittent modalities that are more accessible, and monitor more frequently than the standard 12-lead ECG. Follow-up periods range from 5 days to 3 years. Trials studying photoplethysmography have the shortest follow-up times (5–30 days), followed by those studying ambulatory ECGs (30 days to 2 years), and implantable devices (3 years).

### 3.4. Management

Three-quarters of management studies are randomized (Figure 3). Half of these studies (*n* = 8/16, 50.0%) test rate and/or rhythm control, one-third (*n* = 6/16, 37.5%) examine antithrombotic management, and 12.5% (*n* = 2) monitor for AF recurrence (Table 4). See Table A2 in Appendix A for data on the types of management interventions studied.

The follow-up periods of the studies vary depending on their primary outcome. Studies focusing on cardioversion have the shortest outcome measurement periods (30 min–4 h), followed by those focused on the maintenance of sinus rhythm (2–3 days), and then studies looking at stroke and bleeding risk (2–3 years). The Subclinical POAF (POAF-ILR) trial (NCT02522364) is the only ongoing study looking for recurrent AF in the long-term (2 years after discharge) [20]. Two large (>1000 participant) trials are ongoing. IRCT20200304046696N1 is investigating stroke, transient ischemic attack (TIA), and major bleeding up to 30 days after discharge in 1590 patients with POAF randomized to warfarin or rivaroxaban [21]. The PACES trial (NCT04045665) is looking at a composite of death, stroke, TIA, myocardial infarction, and thromboembolic events up to 90 days post randomization in 3200 patients who developed POAF and are randomized to antiplatelets alone or antiplatelets in combination with oral anticoagulation [22]. Both of these large studies are restricted to patients who underwent CABG.

## 4. Discussion

POAF in cardiac surgery patients is an active field of research, where most studies continue to test prevention strategies. A minority of studies investigate the prediction, detection, and management of POAF. Only a few studies are anticipated to affect clinical practice, and large care gaps remain uninvestigated.

The prediction of POAF continues to be an area of intense research. The mechanisms and pathogenesis of POAF remain poorly understood [23]. While previous studies have developed and internally validated risk models for POAF, no models are recommended by guidelines or widely used in clinical practice [24,25]. This may be related to issues with model performance, ease of use, and uncertainty regarding whether using a prediction model will change management and outcomes. About three quarters of the ongoing studies in this domain aim to identify individual factors that may increase the risk of POAF and could identify targets for future interventional studies. Research has shown that prediction models rarely make it into clinical practice [26]. In order to be adopted into clinical practice, new prediction tools need to be inexpensive, readily accessible, and widely generalizable. It seems unlikely that the bulk of the studies that are currently underway will meet these criteria and ultimately contribute to patient care.

Prevention studies account for nearly two-thirds of studies that are underway. A 2013 Cochrane review identified 118 randomized trials with 138 treatment groups and 17,364 participants and found that each of amiodarone, beta-blockers, sotalol, magnesium, atrial pacing, and posterior pericardiotomy were effective for reducing POAF [27]. Research continues both with these therapies and with interventions that have been disproven (e.g., steroids) [28]. Clinical practice guidelines recommend beta-blockers, amiodarone, and other therapies to prevent POAF. However, surveys and real-world data show that despite evidence and guidelines, these therapies are not provided to a large number of cardiac surgery patients [29]. It is unclear whether this underuse relates to limitations of existing therapies or a problem with knowledge translation. This review identified several trials involving pharmacologic therapies and intra-operative interventions, including newer classes of antiarrhythmic medications (e.g., landiolol and alternate forms of delivery). Whether these therapies will be safe, effective, affordable, and accessible enough to be widely implemented in practice remains unknown.

Guidelines vary in their recommendations for either rate or rhythm control for patients who develop POAF. A recent systematic review and meta-analysis by our group concluded that limited low-quality evidence guides this research question [30]. Ongoing studies in this area have follow-up periods of only 2–3 days post-operatively, and are unlikely to strengthen the body of evidence for this clinical problem.

A knowledge gap remains with respect to when and how to monitor POAF recurrence after hospital discharge. Opportunistic screening may miss the paroxysmal nature of POAF recurrence. In contrast, continuous monitoring is costly and may identify some individuals with a low-risk, low-burden AF. In a systematic review, we found that approximately a third of patients with POAF have AF recurrence detected by an implantable loop recorder in the first year after surgery [31]. Two small studies with 100–150 patients are testing implanted monitors and wearable technologies to detect POAF recurrence over follow-up times of 1 to 2 years [20,32]. Patients and clinicians need recurrence monitoring studies like these on a larger scale, testing these devices in more diverse patient populations and linking them to adverse outcomes.

Ischemic stroke may be the most devastating complication of POAF, and evidence is needed to guide oral anticoagulation in this population. Four ongoing randomized trials are investigating oral anticoagulants and their effect on stroke, TIA, and bleeding risk in patients with POAF. The large, randomized PACES trial is testing an anticoagulation strategy versus a no-anticoagulation strategy and is limited to patients with isolated CABG [22]. This study may change practice for the roughly 50% of cardiac surgery patients who undergo isolated CABG, but clarifying the role of anticoagulation for the other half of patients who undergo other types of cardiac surgery will remain an urgent and unmet need [33].

In summary, the current research landscape for POAF is dominated by studies that are either small, redundant, or address low-priority questions and are unlikely to change clinical practice. Only a small number address large knowledge gaps and areas of high need. Research resources are scarce, and we have an ethical responsibility to research subjects to invite them to participate only in research that is potentially valuable.

The findings of this review are relevant to researchers, funding bodies, professional societies, and clinicians. Before embarking on a new study in the POAF field, researchers should ensure that the clinical question is not answered by existing literature and unlikely to be answered by research that is already in progress [34]. Funding bodies may recognize persistent gaps (e.g., rate/rhythm control and long-term follow-up of POAF patients) and tailor funding calls to address them. The international cardiac surgical community should gather, identify research priorities, and collaborate to answer these questions definitively. Presently, no major society produces focused guidelines on POAF. Clinical practice guidelines that are specific to POAF after cardiac surgery may help with knowledge translation for those who care for patients and those who design and fund studies.

This review has several limitations. Each trial registry has a different set of information that is presented in a unique way. For instance, none of the records in the IRCT or European Union Clinical Trial Registry have been updated since they were created, so it is possible that we excluded some studies registered prior to 2017 that are still ongoing. Additionally, ChiCTR records do not include a timeframe for outcome measurement. Even within a registry, the records vary in their level of detail, so the data we were able to extract may not reflect the totality of data in the trials. Finally, we acknowledge that many nonrandomized studies are not registered and would not have been captured by this review.

## 5. Conclusions

More than one hundred ongoing studies are investigating methods to predict, prevent, detect, and manage POAF after cardiac surgery. Prediction studies produce models that may be useful in clinical practice. The largest number of studies test new modalities for prevention, many of which are randomized trials, that may identify new therapies for preventing AF. One large, randomized trial evaluates the role of anticoagulation, but restricts enrolment to patients who have undergone isolated CABG. Two major clinical care gaps that will persist based on ongoing research are the acute rate and rhythm management of patients with POAF and defining their long-term follow-up. Future randomized trials should compare rate and rhythm control for patients with POAF, and long-term follow-up studies are needed to investigate different approaches to monitoring for AF recurrence in patients with POAF. We need coordinated and systematic efforts to ensure that future research in this field can address clinical questions that are answerable and relevant.

## Figures and Tables

**Table 1:** Primary variables in studies on prediction of POAF in cardiac surgery patients.

Category	Examples	*n* * (% Predictive Studies)
Atrial imaging	Magnetic resonance imaging, 2D echocardiography, computed tomography, fibrosis patterns	9 (42.9)
Biomarkers	Inflammatory cytokines, fibrotic markers, lactate, aldosterone, homocysteine, endothelin-1, myeloperoxidase, brain natriuretic peptide, pro-collagen-1, calcitonin	6 (28.6)
Electrocardiography	P-wave characteristics, signal-processed surface electrocardiography	3 (10.0)
Demographic and past medical data	Risk factors (diabetes, hypertension, smoking), previous cardiac interventions, preoperative cardiac status, preoperative medication, type of operation, post-operative events, discharge	3 (14.3)
Gene expression	Atrial myocyte transcription and translation, IL-6 genotype	2 (9.5)
Other	Perioperative characterization of autonomic nervous system and microcirculation, perioperative anesthesia management, or not specified	3 (14.3)
Multi-variable **	**NCT05255224 (unspecified multimodal)** **NCT03169608 (perioperative characterization of the autonomic nervous system and microcirculation)** **ChiCTR-OCH-13003050 (genotype, serum biomarkers, ECG characteristics)** **CTRI/2022/11/047778 (ECG P-wave characteristics, imaging for left atrial strain)** NCT03466125 (demographics, medical history, post-op events) NCT03962166 (electrocardiogram and echocardiography) NCT00614107 (atrial fibrosis and biomarkers) JPRN-UMIN000013673 (computed tomography of epicardial adipose, inflammation biomarkers)	8 (38.1)

* If a single study plans to investigate *x* unique predictive categories, it counted *x* times. ** Multi-variable represents the predictive studies that test multiple predictive categories. Studies in the multi-variable category are also accounted for in the individual predictive categories above. **Bolding** denotes studies that focus on creating predictive models.

**Table 2:** Interventions in studies of the prevention of POAF in cardiac surgery patients.

Category	Examples	*n* * (% Prevention Studies)
Beta-blockers	*Bisoprolol*, *metoprolol*, *landiolol*, *carvedilol*	11 (14.3)
Antiarrhythmic	*Amiodarone*, *dronedarone*, *sotalol*	11 (14.3)
Vitamin and electrolyte supplementation	*Magnesium sulfate*, *Vitamin C*, *selenium*, *potassium*, *tocotrienols*	11 (14.3)
Autonomic nervous system modulation	*Calcium autonomic denervation*, *stellate ganglion block*, *ganglionic plexi cryoablation*, *vagus nerve stimulation*	11 (14.3)
Anesthetics/analgesics	*Lidocaine*, *ropivacaine*, low opioid protocols, *dexmedetomidine*	8 (10.4)
Paralytic	*Botox*	6 (7.8)
Anti-inflammatory	*Hydrocortisone*, *minocycline*, *colchicine*	6 (7.8)
Antihypertensive	*Spironolactone*, *nitroprusside*, metoral	5 (6.5)
Surgical interventions	*Marshall ligament removal*, *Waterstone fat pad removal*, *left atrial appendage resection*, *cavoatrial annulation*, *biatrial cannulation*, *biatrial pacing*	4 (5.2)
Statins	*Atorvastatin*, *rosuvastatin*	4 (5.2)
Ischemic Conditioning	*Ischemic pre- and post-conditioning*	2 (2.6)
Other	*NP202*, *melatonin*, *ivabradine*, *dapagliflozin*, *Nux Vomica*, *anthocyanin-rich flour*, clinician visits, *human amniotic membrane*	8 (10.4)

* If a single study plans to investigate *x* unique active intervention categories, it counted *x* times. *Italicization* denotes interventions that are being investigated in randomized trials.

**Table 3:** Characteristics of studies examining detection modalities for POAF after cardiac surgery.

Category	Modality	Follow-Up Duration	*n* (Expected) Enrolment	Recruitment Status
Intermittent ECG	Single-lead and 4-lead ECG	3 months post-op	730	Recruitment complete
	Handheld thumb ECG	2 years post-op	250	Recruitment complete
Continuous ECG	Implantable loop recorder	3 years post-op	52	Recruitment complete
	BraveHeart wearable ECG	30 days post-discharge	100	Actively recruiting
Photoplethysmography	ScanWatch	5 days post-op	358	Actively recruiting
	Fibricheck application	30 days post-op	300	Not yet recruiting
Artificial intelligence	Artificial intelligence-assisted AF recognition	Not specified	660	Actively recruiting

**Table 4:** Characteristics of studies of patients who develop POAF after cardiac surgery.

Study Design	Theme and Registry ID	Intervention; Comparator(s)	Primary Outcome(s)	N (Expected) Enrolment	Recruitment Status
Randomized Experimental	Rate or Rhythm Control ChiCTR2000033860	Nifekalant; amiodarone	Cardioversion	100	Actively recruiting
	Rate or Rhythm Control NCT04748991	Vernakalant; amiodarone	Cardioversion	50	Not yet recruiting
	Rate or Rhythm Control NCT05169866	Nifekalant; amiodarone	Cardioversion	274	Not yet recruiting
	Rate or Rhythm Control ChiCTR2000039611	Nifekalant; amiodarone	Maintenance of sinus rhythm	100	Actively recruiting
	Rate or Rhythm Control NCT04223739	Landiolol and oral bisoprolol; infused and oral amiodarone	Maintenance of sinus rhythm	380	Unknown (last known to be recruiting)
	Rate or Rhythm Control NCT03525860	Acupuncture; standard of care	Number of patients that complete acupuncture	40	Recruitment complete
	Antithrombotic Strategies NCT03702582	Rivaroxaban; warfarin	Post-operative length of stay	300	Actively recruiting
	Antithrombotic Strategies NCT05300555	Rivaroxaban; warfarin	Cost effectiveness via QALY	50	Actively recruiting
	Antithrombotic Strategies NCT04045665	Antiplatelet only; oral anticoagulant and antiplatelet	Composite of death, stroke, TIA *, MI *, and systemic arterial/venous thromboembolism	3200	Actively recruiting
	Antithrombotic Strategies IRCT20200304046696N1	Rivaroxaban; warfarin	Stroke, TIA *, and major bleeding	1590	Actively recruiting
	Antithrombotic Strategies JPRN-UMIN000037139	Apixaban; edoxaban	Bleeding and thromboembolic events	200	Terminated (not actively recruiting)
	Monitoring for AF Recurrence NCT02522364	Implantable loop recorder for 7 years; biannual clinician visit with Holter at 3 and 6 months	AF recurrence, MACCE *, implantation of pacemaker or ICD *, device complications, and major bleeding	150	Unknown (last known to be recruiting)
Nonrandomized Experimental	Antithrombotic Strategies JPRN-UMIN000021138	Edoxaban	Stroke, TIA *, and major bleeding	150	Actively recruiting
Observational	Rate or Rhythm Control NCT04804748	Low-energy cardioversion w/biatrial pacing; w/o pacing	Cardioversion and POAF recurrence.	450	Not yet recruiting
	Rate or Rhythm Control NCT05165862	Beta-blockers; digoxin; amiodarone	Maintenance of HR < 110 bpm	54	Recruitment complete
	Monitoring for AF Recurrence NCT05664308	Only ECG; ECG and photoplethysmography monitoring	Recurrence rate of AF	106	Not yet recruiting

* MACCE = major adverse cardiac events: MI = myocardial infarction; TIA = transient ischemic attack; ICD = implantable cardioverter–defibrillator.

## Data Availability

The study database is made up entirely from publicly available sources. Data are available upon reasonable request to the corresponding author.

## Appendix A

**Table A1:** Characteristics of ongoing studies of POAF after cardiac surgery.

	Ongoing Trials *n* = 121
**Study theme**	
Prediction (%)	21 (17.4)
Prevention (%)	77 (63.6)
Detection (%)	7 (5.8)
Management (%)	16 (13.2)
**Study design**	
Randomized experimental (%)	82 (67.8)
Nonrandomized experimental (%)	13 (10.7)
Observational (%)	26 (21.5)
**Trial registry**	
Clinicaltrials.gov (%)	68 (56.2)
IRCT (%)	17 (14.0)
ChiCTR (%)	11 (9.1)
UMIN-CTR (%)	9 (7.4)
CTRI (%)	5 (4.1)
Dutch trial registry (%)	4 (3.3)
EUCTR (%)	4 (3.3)
ANZCTR (%)	2 (1.7)
ISRCTN (%)	1 (0.8)

**Table A2:** Interventions and comparators of management studies in ongoing studies on POAF after cardiac surgery.

Classes of Interventions	Examples	*n* * (% Management Studies)
Antiarrhythmic	*Amiodarone*, *nifekalant*, *vernakalant*	6 (37.5)
Anticoagulant	*Warfarin*, *rivaroxaban*, *edoxaban*, *apixaban*	5 (31.3)
Beta-blockers	Landiolol, bisoprolol, metoprolol	2 (12.5)
Recurrence monitoring	*Implantable loop recorder*, photoplethysmography	2 (12.5)
Rate controlling	Digoxin	1 (6.3)
Cardioversion	Low-energy cardioversion	1 (6.3)
Pacing	Biatrial pacing	1 (6.3)
Other	*Antiplatelets*, *acupuncture*	2 (12.5)

* If a single study plans to investigate *x* unique management categories, it is counted *x* times. *Italicization* denotes interventions that are being investigated in randomized trials.
